# Stacked optical antennas for plasmon propagation in a 5 nm-confined cavity

**DOI:** 10.1038/srep11237

**Published:** 2015-06-09

**Authors:** A. Saeed, S. Panaro, R. Proietti Zaccaria, W. Raja, C. Liberale, M. Dipalo, G. C. Messina, H. Wang, F. De Angelis, A. Toma

**Affiliations:** 1 Istituto Italiano di Tecnologia, via Morego 30, I-16163 Genova, Italy; 2 Università degli Studi di Genova, 16145 Genova, Italy

## Abstract

The sub-wavelength concentration and propagation of electromagnetic energy are two complementary aspects of plasmonics that are not necessarily co-present in a single nanosystem. Here we exploit the strong nanofocusing properties of stacked optical antennas in order to highly concentrate the electromagnetic energy into a 5 nm metal-insulator-metal (MIM) cavity and convert free radiation into guided modes. The proposed nano-architecture combines the concentration properties of optical nanoantennas with the propagation capability of MIM systems, paving the way to highly miniaturized on-chip plasmonic waveguiding.

Strongly coupled plasmonic nanosystems have unveiled outstanding scenarios in the sub-wavelength confinement of electromagnetic (EM) fields (*i.e.* hot-spots generation^1^,^2^,^3^), finding applications in ultrasensitive Raman^4^ and Infrared^5^ detection down to the single/few-molecules regime^6^. Moreover, the plasmonic enhancement of EM fields has been widely exploited for the promotion of intrinsically weak nonlinear effects^7^,^8^. A natural step ahead with respect to EM nanofocusing consists in the guided EM energy delivery at the nanoscale, opening novel perspectives in the on-chip integration of nano-photonic circuits^9^, all-optical gates^10^ and nanodetectors^11^. Among the systems supporting propagating modes, we can mention plasmonic waveguides^12^, adiabatic nanocones^13^,^14^,^15^ and hollow nano-cylinders^16^.

A particular category of plasmonic waveguides consists in *metal-insulator-metal* (MIM) cavities^17^, which can concentrate EM energy in ultrasmall volumes without theoretical cut-off limitations^18^. In literature MIM cavities have been extensively investigated as high-quality resonators supporting several typologies of confined and localized modes^19^,^20^,^21^ with remarkable Purcell factors^22^. Within this context, Choo *et al.* have recently realized a 3D tapered MIM cavity that efficiently funnels the incoming radiation into a strongly confined MIM waveguide, by means of a quasi-adiabatic energy transfer from micro- to nano-scale^18^.

In concomitance with the conceiving of 3D tapered MIM cavities, also planar nanoantenna-based devices have been studied for the optimal coupling of free radiation to plasmonic guided modes^23^. The localized surface plasmons (LSPs) supported by nanoantennas can be directly excited by far-field radiation, guaranteeing the EM energy focusing necessary for sub-wavelength energy delivery. The possibility to combine the EM coupling properties of nanoantennas with the EM propagation capability of MIM cavities could lead to significant advantages in the on-chip miniaturization of plasmonic waveguides.

Here we investigated a 3D stacked optical antennas (SOA) configuration^24^ endowed with different inter-particle cavity separations, able to support transverse electric and magnetic (TEM) guided modes. By combining spectroscopic measurements and simulative approaches we demonstrated that the coupling strength between the free radiation and the propagating cavity modes presents a dependency on the illumination angle, mainly related to the alignment between the electric fields outside and inside the cavity. Finally, in optimal coupling configuration, we experimentally observed how the guided modes resonances are preserved even in the case of 5 nm thick SOA cavity.

## Results

**Figure 1:**
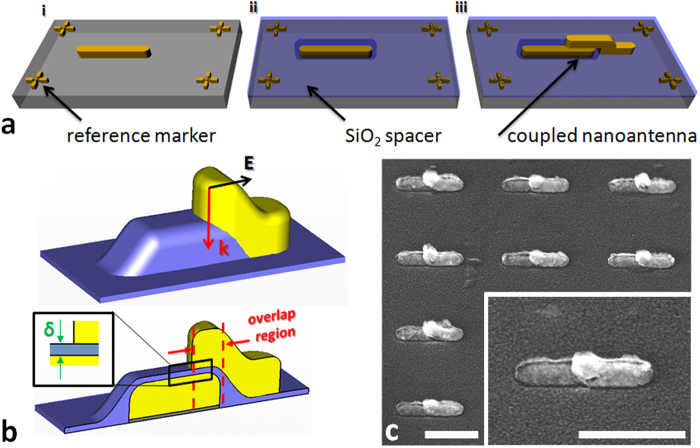
Stacked Optical Antennas design. **a**, sketches reporting the overlayer exposure fabrication steps: i) underlying structure patterning (60 nm gold on CaF_2_ substrate); ii) dielectric spacer deposition (9 nm SiO_2_); iii) overlayer patterning (60 nm gold on SiO_2_ layer). **b**, respectively outer (upper sketch) and inner (lower sketch) geometry of a single SOA assembly. The lower sketch is the profile of a SOA on a plane that passes through the center of the structure and is perpendicular to its short axis. **c**, SEM cut-out of SOAs array (Inset: close-up on a single SOAs assembly) (scale bars: 300 nm).

We fabricated SOA arrays by a two-step electron beam lithography (EBL) procedure (see “Fabrication” sub-section in Supplementary Information, section 1): i) we patterned an array of planar nanoantennas on a CaF_2_ substrate, inserting reference markers close to the structures (step 1 in Fig. 1a); ii) we deposited a layer of SiO_2_, as dielectric spacer, on top of the array by means of thermal evaporation (step 2 in Fig. 1a); iii) finally, we exploited the previous markers as aligning references and we fabricated a new nanoantenna array on the SiO_2_ layer (step 3 in Fig. 1a), so that lower and upper nanoantennas resulted only partially overlapped (see overlap region in lower part of Fig. 1b). This protocol allows to fabricate reproducible arrays of SOAs with inter-particle separation (defined *δ,* as reported in the inset of Fig. 1b) that can be pushed down to the thickness accuracy of the SiO_2_ deposition process (Fig. 1c).

As it can be appreciated in the lower sketch of Fig. 1b, we have been able to create a nanoantenna dimer system that presents a MIM cavity, defined by the partial overlapping of the two antennas.

**Figure 2:**
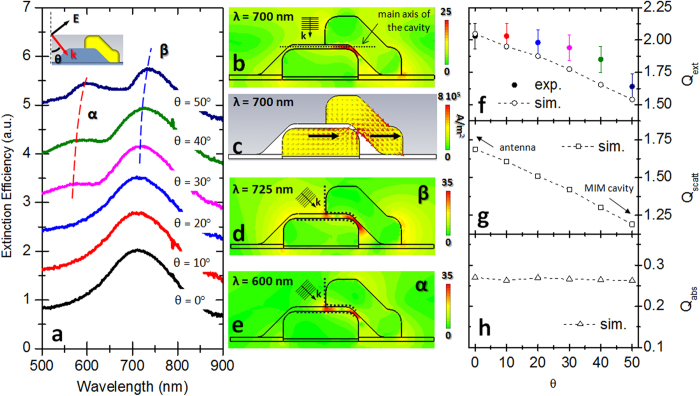
SOAs optical characterization. **a**, extinction efficiency spectra of SOAs for varying light incidence angles. **b,c**, 2-D plots simulated, in resonance condition (700 nm), on a plane that passes through the center of the system, perpendicular to the SOA short axis: respectively normalized electric field distribution and charge density distribution. **d,e**, normalized electric field distributions respectively of the *β* and *α* resonances for *θ* = 50°. **f-h**, respectively extinction (*Q*_*ext*_), scattering (*Q*_*scatt*_) and absorption (*Q*_*abs*_) efficiencies of SOAs in correspondence of the peak around 700 nm, as a function of the light incidence angles.

We performed transmission spectroscopy on SOAs with *δ* = 9 nm for incoming light normal to the substrate and polarization parallel to the long axis of the system (see upper sketch in Fig. 1b) (see “Optical characterization” sub-section in Supplementary Information, section 1). The corresponding extinction efficiency spectrum is reported in Fig. 2a (black curve) revealing an intense peak centered around 700 nm. The extinction efficiency is defined as *Q*_*ext*_ = *A*(1-*T*_*rel*_)/*Na*, where *A* is the total area illuminated by the light source, *T*_*rel*_ is the relative transmittance of the sample, *a* is the area of a single SOAs assembly and *N* is the number of SOAs illuminated by the light spot. By means of finite-integration code software (for more details on the simulations performed, see Supplementary Information, section 2) we numerically investigated the normalized electric field (defined as the ratio between the local and the incoming field 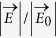) distribution inside the SOAs (the simulation design has been realized basing on a cross-sectional investigation of the fabricated samples. For more details see the “Cavity design” sub-section in Supplementary Information, section 1). In resonance condition (700 nm), we noticed how the local response of the device is characterized by the arising of an intense hot-spot in the middle of the cavity (Fig. 2b). From the corresponding charge current density distribution (Fig. 2c), we can observe that the SOAs react to normal incident EM radiation as a planar nanoantenna dimer. In fact the LSPs in both antennas can be associated to parallel dipolar momenta oscillating in phase (black arrows in Fig. 2c). Such EM behavior corresponds to the less favorable condition for the excitation of MIM cavity modes. In fact, in normal incidence condition, the wave-vector “
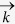
” associated to the incoming radiation is perpendicular to the principal axis of the cavity (see Fig. 2b). As a consequence, the incident electric field couples to the structure without transferring EM moment along the cavity and a static hot-spot is generated in the middle of the cavity. In section 3 of Supplementary Information we reported the Poynting vector distribution inside the cavity for *λ* = 700 nm.

In order to investigate the coupling between the free radiation and the MIM cavity modes, we performed transmission spectroscopy by varying the light incidence angle *θ* from 0° to 50°, at steps of 10°, with respect to the normal incidence condition (see upper sketch in Fig. 2a) (The same investigation has been conducted on *δ* = 20 nm SOAs, as we report in “Cavity design” sub-section of Supplementary Information, section 1). From the corresponding extinction efficiency spectra (Fig. 2a), no appreciable variations can be noticed for the 10° and 20° cases with respect to the normal incidence condition (*θ* = 0°). A significant change instead occurs in the 30° line-shape (pink curve), where a bimodal response can be clearly appreciated. At larger *θ*, we observe an intensification of the high energy peak *α* and an overall red-shift of the extinction efficiency spectra (as it can be appreciated from the low energy peak *β*). Finally, for *θ* = 50° condition, we observed *α* peak around 600 nm and *β* peak around 725 nm. We simulated the near-field response of the system for *θ* = 50° incidence angle respectively in *α* and *β* resonance condition and we reported the corresponding near-field distributions in Fig. 2d,e. As it can be appreciated in both the plots, the free radiation in out-of-normal incidence condition induces the excitation of different plasmonic resonances with respect to Fig. 2b.

In concomitance with the evolution of the near-field response, also the far-field observables of SOAs present a significant change as a function of the light incidence angle. In particular, focusing on the extinction efficiency peak around 700 nm (spectra of Fig. 2a), we observe how the associated intensity decreases of the 20% accordingly to the rise of *θ* from 0° to 50° (see full dots in Fig. 2f). This far-field behavior can be ascribed to the gradual coupling between free radiation and guided modes. Since plasmonic antennas are optimal scattering systems, they exhibit high extinction efficiency values in correspondence of their plasmon resonance. As a consequence, the decrease of the extinction efficiency indicates a remarkable modification in the far-field properties of a plasmonic system evolving from a pure nanoantenna to a waveguide cavity. In order to confirm these assumptions, we reported the simulated intensities of the corresponding peaks, as a function of *θ* (empty dots in Fig. 2(f)). It is worth noticing that the SOAs scattering efficiency undergoes a significant reduction in concomitance of the *θ* increase (see Fig. 2g). At the same time, the absorption efficiency of SOAs does not show a remarkable evolution with *θ* (Fig. 2h), suggesting that the phenomenon under study is strictly related to the scattering properties of the system. This far-field evolution is therefore compatible to the coupling between free radiation and guided modes inside SOAs, in out-of-normal incidence condition. By increasing *θ*, the system gradually behaves like a MIM cavity rather than a simple nanoantenna dimer.

**Figure 3:**
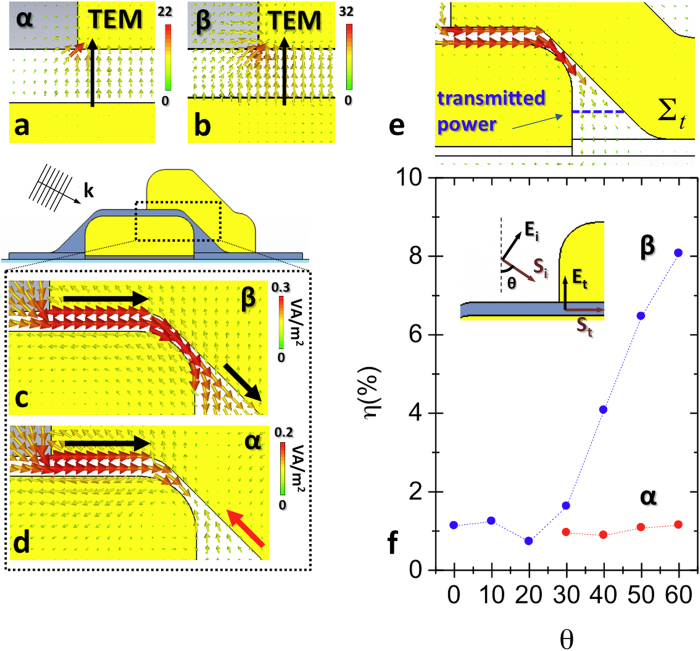
Propagating mode analysis. **a,b**, vectorial electric field distributions respectively of *α* and *β* configurations along the MIM cavity. **c,d**, Poynting vector distributions respectively of *β* and *α* configurations along the MIM cavity. **e**, sketch depicting the profile of the surface (blue lines) on which the transmitted power associated to *β* and *α* resonances has been evaluated. **f**, plot reporting the ratio *η* between the power transmitted inside the cavity (*P*_*t*_) and the impinging power (*P*_*0*_) as a function of the light incidence angle, in *β* and *α* resonance condition.

In order to better characterize the *α* and *β* configurations, we analyzed the vectorial distribution for the electric fields (Fig. 3a,b) and the Poynting vector 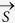
(Fig. 3c,d) along the MIM cavity in the two resonant conditions for *θ* = 50°. In both the cases, the electric field is mainly perpendicular to the interface between the plasmonic antennas and the SiO_2_ spacer. In section 4 of Supplementary Information we show that the magnetic field associated to *α* and *β* resonances remains perpendicular both to 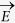 and 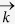 along the cavity, suggesting the TEM nature of the propagating modes considered.

By plotting the Poynting vector distribution associated to the *β* resonance (Fig. 3c), we can recognize a guided mode travelling along the MIM cavity from left to right (see black arrows). Conversely, in *α* condition (Fig. 3d), we can notice a propagating wave from left to right (black arrow) and a back-reflected (from right to left) wave (red arrow) that converge towards the center of the cavity. The presence of propagating modes implies that a fraction of the incoming EM energy can be resonantly funneled inside the SOAs cavity.

In order to quantify the funneling efficiency of the SOAs assembly, we evaluated the power (*P*_*0*_) impinging on the system (for more details see Supplementary Information, section 5) and the power transmitted inside the sub-10 nm cavity (*P*_*t*_). The transmitted power has been calculated by the flux of 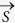 on a surface inside the cavity (∑_*t*_) (see Fig. 3e). By plotting in Fig. 3f the funneling efficiency *η* (*P*_*t*_/*P*_*0*_) associated to “*β*” resonance (blue dots) as a function of the incidence angle *θ*, we could appreciate how the corresponding guided mode practically does not couple to free radiation for small incidence angles (see *θ* < 30°). By increasing *θ*, the energy funneled inside the cavity starts increasing and *η* reaches an 8% value for *θ* = 60°. The raise of the coupling strength between free radiation and TEM cavity modes as a function of *θ* can be essentially ascribed to the gradual alignment of the incoming light polarization (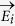) with the electric field direction inside the MIM cavity (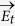) (see the sketch in Fig. 3f). On the contrary, the *η* parameter associated to “*α”* resonance does not show an appreciable evolution for 30° < *θ* < 60° (remains almost constant around 1%), because of the counter-propagating wave which opposes the forward energy flux.

**Figure 4:**
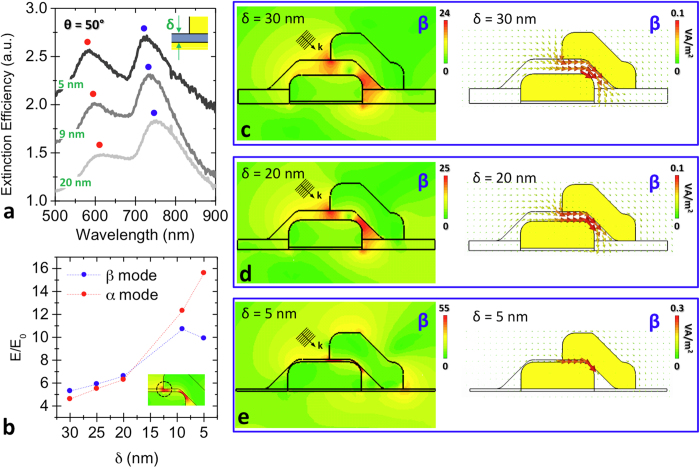
Sub-10 nm EM confinement in SOAs. **a**, experimental extinction efficiency spectra of SOAs with 20 nm, 9 nm and 5 nm thickness SiO_2_ spacer, collected for 50° incidence illumination. **b**, plot reporting the normalized electric field 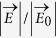 at the left extremity of the cavity (see inset) for 50° light incidence angle, as a function of the spacer thickness, in *β* and *α* resonance condition. **c-e**, normalized electric field (left plots) and Poynting vector distributions (right plots) of the *β* configuration inside the MIM cavity, at 50° light incidence condition, for three different spacer thicknesses: respectively 30 nm, 20 nm and 5 nm.

For experimental reasons, the maximum light incidence angle of our optical set-up was limited to *θ* = 50°. Therefore, in the best coupling conditions we could achieve, we varied the inter-particle cavity size “*δ*” in order to analyze the energy confinement capability of the MIM device. In particular, we collected the *θ* = 50° extinction efficiency spectra for SOA arrays with *δ* = 20 nm, 9 nm and 5 nm (Fig. 4a). As we can notice, the decrease in the spacer thickness induces a significant intensification of the *α* peak, while the *β* peak remains substantially constant.

In order to interpret the evolution of the extinction efficiency spectra as a function of *δ*, we considered the normalized electric field 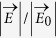 distribution around the left extremity of the SOAs cavity. The incidence angle of the exciting source was fixed at 50° and *δ* was varied between 30 nm and 5 nm. The normalized electric field values have been plotted, both for *β* (blue dots) and *α* resonance (red dots), in Fig. 4b as a function of the decrease in the SiO_2_ layer thickness. As we can notice, the local field associated to the *β* and *α* resonances moderately varies for *δ* > 20 nm, due to the increase in the cavity confinement. For smaller thicknesses (*δ* < 20 nm), the field enhancement of *β* resonance saturates around 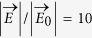, in accordance to the steadiness of *β* peak intensity reported in Fig. 4a. On the other hand, the *α* resonance behavior presents a strong increase for the lower thickness values. This field intensification can be ascribed to a higher difficulty of the *α* distribution to propagate inside the cavity, leading to an almost localized hot-spot. The corresponding extinction efficiency peak coherently increases (see *α* peak in Fig. 4a) even if it is not associated to an efficient propagating mode. In order to confirm that the *β* resonance is sustained along sub-10 nm thickness cavities, we reported in Fig. 4c–e both the electric field enhancement and the Poynting vector distributions respectively for 30 nm, 20 nm and 5 nm thick cavities. In the left plots it is possible to appreciate the gradual confinement experienced by the EM radiation interacting with SOA nanoassembly. Finally, in the right plots, it is shown how the propagating *β* resonance is preserved even along the 5 nm-confined cavity.

## Conclusion

We presented a SOA device able to efficiently funnel free EM radiation into a 5 nm size cavity, exciting different orders of plasmonic guided modes. The induction of TEM propagating modes inside the MIM cavity has been observed by optical spectroscopy and analyzed *via* EM simulation approach. By varying the incidence angle of the incoming radiation, we have been able to find an illumination configuration that guarantees an appreciable coupling (*η* = 8%) of the free EM waves to the plasmonic guided modes supported by the device. Finally, due to the high reproducibility of the fabrication technique employed and to the fine control on the sub-wavelength cavity thickness obtained, SOA system can be considered as an ideal candidate for the implementation of on-chip devices sustaining highly localized light propagation.

## Methods

### Fabrication

Stacked optical antenna (SOA) matrices have been fabricated recurring to a two-step Electron Beam Lithography (EBL) nanopatterning procedure. After substrate-cleaning in an ultrasonic bath of acetone, PolyMethylMethacrylate (PMMA) electronic resist has been spin-coated on the substrate at 1800 rpm. Hence, annealing has been performed at 180 °C for 7 min in order to obtain a uniform film. In perspective of preventing surface charging and drift effects, 10 nm Al layer has been thermally deposited on the PMMA surface. Therefore EBL machine (electron energy 20 KeV and beam current 45 pA), equipped with a pattern generator (Raith 150-two), has been employed for the nanostructure patterning. Once terminated such procedure the Al layer has been removed in a KOH solution and then the exposed resist was developed in a conventional solution of MIBK/isopropanol (IPA) (1:3) for 30 s. Physical Vapour Deposition (evaporation rate 0.3 Å/s) respectively of 3 nm Ti as adhesion layer and 60 nm Au has been performed on the sample. Finally, the unexposed resist was removed in ultrasonic bath of acetone and the sample has been rinsed out in IPA. Following this protocol, we patterned an array of planar nanoantennas on a CaF_2_ substrate, inserting reference markers close to the structures. We deposited a layer of SiO_2_, as dielectric spacer, on top of the array by means of thermal evaporation. Finally, we exploited the previous markers as aligning references and we fabricated a new nanoantenna array on the SiO_2_ layer, so that lower and upper nanoantennas resulted only partially overlapped.

### Optical characterization

The optical properties of the SOAs have been analyzed by far-field transmission spectroscopy in a range between 500 nm and 900 nm. In order to collect appreciable far-field signals from the plasmonic nanostructures, 40 μm × 40 μm size matrices of SOAs were patterned on CaF_2_ (100) substrate, employed for its high transparency in visible (VIS) and near-infrared (NIR) region. During the optical characterization, the samples have been illuminated at different incidence angles (*θ*) with a linearly polarized VIS-NIR (DH-2000-BAL lamp, Ocean Optics) light source, performing optical spectroscopy (HR4000, Ocean Optics) for polarization parallel to the SOA long axis. The optical set-up employed is the same described in Panaro S. *et al.,* ACS Photonics, 1(4), 310-314, (2014) (Supplementary Information, section 3). The sample has been placed on a rotating stage which allowed the spectroscopic investigation at tilted incidence angle.

## Additional Information

**How to cite this article**: Saeed, A. *et al.* Stacked optical antennas for plasmon propagation in a 5 nm-confined cavity. *Sci. Rep.* **5**, 11237; doi: 10.1038/srep11237 (2015).

## Supplementary Material

Supplementary Information
