# Supplementary material for: Stacked optical antennas for plasmon propagation in a 5 nm-confined cavity
Source: Sci Rep. 2015 Jun 9;5:11237. doi: 10.1038/srep11237 (PMC4460891; doi:10.1038/srep11237)
Supplement: Supplementary Information [file srep11237-s1.doc]

**Stacked optical antennas for plasmon propagation in a 5 nm-confined cavity**

A. Saeed^1,2,†^, S. Panaro^1,2,†^, R. Proietti Zaccaria^1^, W. Raja^1,2^, C. Liberale^1,§^, M. Dipalo^1^, G. C. Messina^1^, H. Wang^1,2^, F. De Angelis^1^, A. Toma^1,^*

^1^ Istituto Italiano di Tecnologia, via Morego 30, I-16163 Genova, Italy.

^2^ Università degli Studi di Genova, 16145 Genova, Italy.

*corresponding author. E-mail address: [andrea.toma@iit.it](mailto:andrea.toma@iit.it)

^†^ these authors contributed equally.

^§^ present address: BESE Division, KAUST, King Abdullah University of Science and Technology, Thuwal, 23955-6900, Kingdom of Saudi Arabia.

**Supplementary Information**

SECTION 1: SAMPLE FABRICATION AND OPTICAL CHARACTERIZATION

SECTION 2: SIMULATIVE ANALYSIS

SECTION 3: POYNTING VECTOR DISTRIBUTION AS A FUNCTION OF *θ*

SECTION 4: INVESTIGATION OF *α* AND *β* RESONANCES

SECTION 5: CALCULATION OF THE INCIDENT POWER (*P_0_*)

**Section 1: Sample fabrication and optical characterization**

**Fabrication**

Stacked optical antenna (SOA) matrices have been fabricated recurring to a two-step Electron Beam Lithography (EBL) nanopatterning procedure. After substrate-cleaning in an ultrasonic bath of acetone, PolyMethylMethacrylate (PMMA) electronic resist has been spin-coated on the substrate at 1800 rpm. Hence, annealing has been performed at 180 °C for 7 min in order to obtain a uniform film. In perspective of preventing surface charging and drift effects, 10 nm Al layer has been thermally deposited on the PMMA surface. Therefore EBL machine (electron energy 20 KeV and beam current 45 pA), equipped with a pattern generator (Raith 150-two), has been employed for the nanostructure patterning. Once terminated such procedure the Al layer has been removed in a KOH solution and then the exposed resist was developed in a conventional solution of MIBK/isopropanol (IPA) (1:3) for 30 s. Physical Vapour Deposition (evaporation rate 0.3 Å/s) respectively of 5 nm Ti as adhesion layer and 60 nm Au has been performed on the sample. Finally, the unexposed resist was removed in ultrasonic bath of acetone and the sample has been rinsed out in IPA. Following this protocol, we patterned an array of planar nanoantennas on a CaF_2_ substrate, inserting reference markers close to the structures. We deposited a layer of SiO_2_, as dielectric spacer, on top of the array by means of thermal evaporation. Finally, we exploited the previous markers as aligning references and we fabricated a new nanoantenna array on the SiO_2_ layer, so that lower and upper nanoantennas resulted only partially overlapped. To be noticed that no Ti layer is present below the upper antenna, in order to prevent high dissipation processes on the cavity modes.


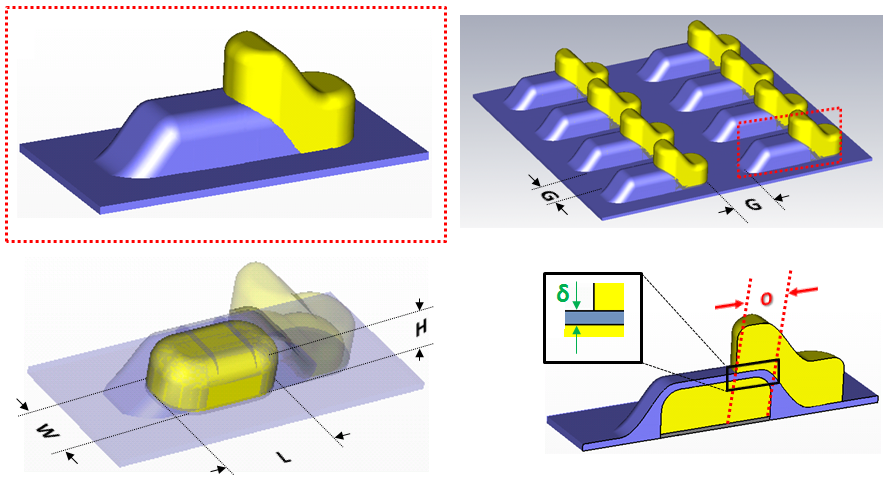


Figure S1.1 Sketches reporting the morphological parameters which describe the SOA arrays fabricated.

According to the labels shown in Figure S1.1, we reported the fabrication parameters of SOAs assembly in the following table:

| L (nm) | W (nm) | H (nm) | *δ* (nm) | O (nm) | G (nm) |
| --- | --- | --- | --- | --- | --- |
| 150 ± 10 | 90 ± 10 | 60 | 5-9-20 | 55 ± 10 | 100 ± 10 |

**Cavity design**

In order to properly design the MIM cavity in the simulation workspace, a cross-sectional investigation of the fabricated SOAs has been performed. Basing on the SEM micrograph depicted in Figure S1.2(a), we designed a sloping cavity of SiO_2_ of equivalent thickness, *δ* = 20 nm (Fig. S1.2(b)). Despite the morphological complexity of the nano-assembly under study, the considered model seems to properly reproduce the optical response of the real system. In fact, if we compare the measured (Fig. S1.2(c)) and simulated (Fig. S1.2(d)) extinction spectra for different incidence angles *θ*, the representative peaks (*α* and *β*) are in good agreement within a reasonable shift between 50 and 100 nm. Nevertheless, by comparing the spectral width of the experimental (Fig. S1.2(c)) and the simulative (Fig. S1.2(d)) spectra, we can recognize a higher broadening of the experimental curves with respect to the simulated ones. Since the system under study is the combination of a nanoantenna dimer and a MIM cavity, we can expect that the discrepancies between measured and simulated spectra present a twofold origin. From one side, the polycrystalline nature of fabricated antennas introduces a broadening of the spectra which can be ascribed to damping effects among grain boundaries. In several works, *e.g.* K.-P. Chen *et al.*, Nano Lett. 10 (2010) 916–922 and S. Panaro *et al.*, Microelectron. Eng. 111 (2013) 91−95, it is shown how annealing processes can promote the partial merging of polycrystalline grains inside plasmonic nanoantennas, inducing a sensible reduction of the internal damping processes and consequently an increase in the spectral sharpness of the plasmonic spectra. On the other hand, the guided modes to which the spectral peaks refer, propagate almost completely through the SiO_2_ spacer. Therefore, the spectral position of the extinction peaks results strongly influenced by the dielectric properties of the cavity. If we consider the thicknesses involved, we can reasonably expect that slight inhomogeneities of the SiO_2_ spacer can be responsible for shifts between measured and simulated spectra.

By looking at the Figure S1.2(c), we can observe that the extinction peak intensity at 700 nm shows a decrease of the 10 % accordingly to the rise of *θ* from 0° to 50° (see full dots in Figure S1.2(e)). This far-field behavior can be ascribed to the gradual coupling between free radiation and guided modes inside SOAs, in out-of-normal incidence condition. By increasing *θ*, the system gradually behaves like a MIM cavity rather than a simple nanoantenna dimer. Since plasmonic antennas are optimal scattering systems, they exhibit high extinction efficiency values in correspondence of their plasmon resonance. As a consequence, the decrease of the extinction efficiency indicates a remarkable modification in the far-field properties of a plasmonic system, which is evolving from a pure nanoantenna to a waveguide cavity. In order to confirm these assumptions, we reported the simulated intensities of the corresponding peaks, as a function of *θ* (empty dots in Figure S1.2(e)). For properly comparing the evolution of the measured and simulated peak intensities, we scaled the simulated values so that the *θ* = 0° extinction peak intensity is equal to the corresponding experimental value. It is worth noticing that the SOAs scattering efficiency undergoes a significant reduction in concomitance of the θ increase (see Figure S1.2(f)). At the same time, the absorption efficiency of SOAs does not show a remarkable evolution with *θ* (Fig. S1.2(g)), suggesting that the phenomenon under study is strictly related to the scattering properties of the system. This far-field behavior is compatible with the onset of guided modes inside the MIM cavity.


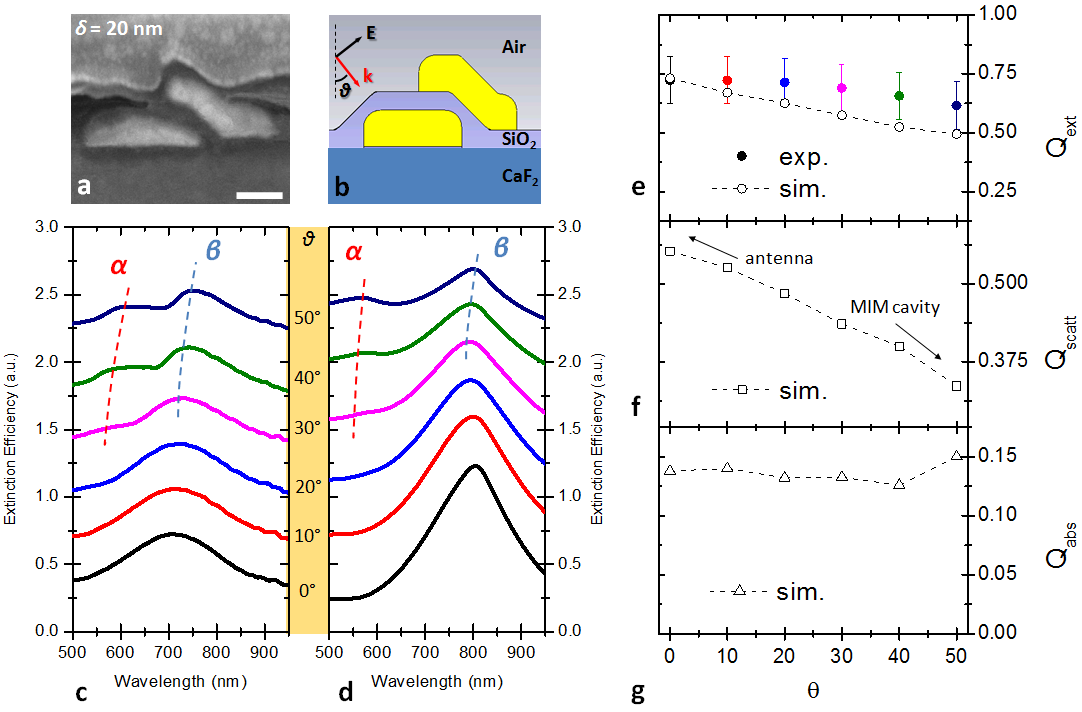


Figure S1.2 (a) Cross-sectional SEM image of a single *δ* = 20 nm SOA nano-assembly (Scalebar: 50 nm). (b) Cavity profile of a SOAs design. (c) Extinction efficiency spectra of SOAs for varying light incidence angles. (d) Simulated extinction efficiency spectra of SOAs for varying light incidence angles. (e-g) Respectively extinction (*Q_ext_*), scattering (*Q_scatt_*) and absorption (*Q_abs_*) efficiencies of SOAs in correspondence of the peak around 700 nm, as a function of the light incidence angles.

**Optical characterization**

The optical properties of the SOAs have been analyzed by far-field transmission spectroscopy in a range between 500 nm and 900 nm. In order to collect appreciable far-field signals from the plasmonic nanostructures, 40 μm x 40 μm size matrices of SOAs were patterned on CaF_2_ (100) substrate, employed for its high transparency in visible (VIS) and near-infrared (NIR) region. During the optical characterization, the samples have been illuminated at different incidence angles (*θ*) with a linearly polarized VIS-NIR (DH-2000-BAL lamp, Ocean Optics) light source, performing optical spectroscopy (HR4000, Ocean Optics) for polarization parallel to the SOA long axis. The optical set-up employed is the same described in Panaro S. *et al.,* ACS Photonics, 1(4), 310-314, (2014) (Supporting Information, Section 2). The sample has been placed on a rotating stage which allowed the spectroscopic investigation at tilted incidence angle.

**Section 2: Simulative analysis**

All the simulations have been carried out by means of a commercial software based on a finite integration code (CST Studio Suite 2010). As shown in the profile sketch of Figure S2(a), the assembly is composed by two gold antennas separated by a layer of SiO_2_. The final design of SOAs, with particular attention on the MIM cavity, has been optimized basing on the cross-sectional analysis shown in section 1. Therefore, the choice and distribution of the materials in CST workspace have been conducted coherently with the fabrication protocol adopted. During the fabrication of the SOAs arrays, a 5 nm Ti layer was evaporated on the CaF_2_ substrate, in order to promote the adhesion of the lower gold antenna to the substrate. For this reason, in the simulation design, a 5 nm layer of titanium has been considered on the bottom of the lower gold antenna (see circled region in Figure S2(a)). On the other hand, no titanium was deposited below the upper antenna in order to prevent high dissipation processes on the cavity modes. Similarly, in the simulation design, no Ti layer has been considered below the upper antenna (see Figure 2(a)), defining a Au-SiO_2_-Au cavity. Since we are expecting that the modes under study will be strongly localized inside the MIM cavity, the effect of the CaF_2_ substrate is not supposed to be critical for the SOAs response. For this reason, the SOAs geometry has been embedded in air. Moreover, due to the strongly confined distribution of the expected plasmonic fields, all the simulations have been conducted on single SOAs assemblies with open boundary conditions.


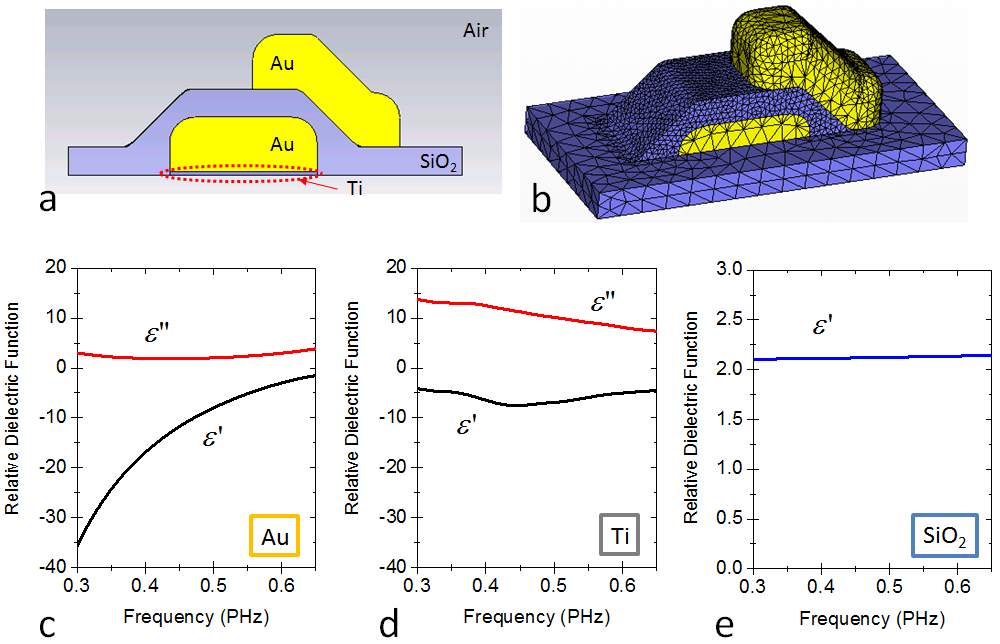


Figure S2. (a) Cavity profile of a SOAs design in the simulative workspace. (b) Representative simulation design in mesh view. (c-e) Relative dielectric functions of respectively gold (c), titanium (d) and silicon dioxide (e) in the spectral region of interest.

The complexity of the SOAs morphology required a dedicated mesh refinement procedure aimed to thicken the number of mesh cells in the spatial regions of higher field gradient. Therefore, we increased the mesh density in the vicinity of the rounded surfaces and inside the SiO_2_ cavity (see Figure S2(b)).

The dispersive nature of the metals employed in our simulations required the proper treatment of the dielectric functions of gold and titanium. The two metals present both real (*ε’*) and imaginary (*ε”*) dielectric functions which, in the spectral region under analysis (visible/near-infrared), can be described *via* a semiquantum Drude-Lorentz relation:

$\hat{\varepsilon}\left(\omega\right)=1-\frac{\Omega_{p}^{2}}{\omega\left(\omega-i\Gamma_{0}\right)}+\sum_{j=1}^{5} \frac{f_{j}\omega_{p}^{2}}{\left(\omega_{j}^{2}-\omega^{2}\right)+i\omega\Gamma_{j}}$ (1)

The material-dependent parameters are tabulated in Rakić A. D. *et al.*, Appl Opt 1998, 37(22), 5271-5283. A plot of both the real and imaginary parts of $\hat{\varepsilon}\left(\omega\right)$ for the two metals has been added respectively in Figures S2(c,d).

The dielectric function of SiO_2_ can be considered constant in the spectral range of interest (see the right plot of Figure S2(e), where the dielectric function has been obtained from Malitson I. H., Appl Opt 1963, 2, 1103-1107). We therefore created a SiO_2_ material with dielectric constant equal to 2.1.

All the simulations have been conducted for plane wave excitation and the amplitude of the incoming electric field amplitude was set to the unity.

**Section 3: Poynting vector distribution as a function of *θ***

In the main text, the mode excited around *λ* = 700 nm in normal incidence condition has been denoted as a non-propagating LSP. In order to confirm this statement, we simulated the vectorial distribution of both the normalized electric field and the Poynting vector associated to the mode inside the cavity. From the electric field plot (Fig. S3(a)) we can deduce that the electric field inside the cavity is mainly perpendicular to the metal-insulator boundaries with a significant intensification in the narrowest part of the cavity. This result is coherent with the charge current density plot reported in Figure 2(c) of the main text. The static nature of the mode under study can be directly verified by observing the associated Poynting vector distribution (Fig. S3(b)) inside the cavity. By increasing the light incidence angle *θ* from 0° to 60° at steps of 10° (Figs. S3(b-h)), it is possible to observe how the *β* resonance starts to be appreciable around *θ* = 30° and its intensity accordingly increases with the incidence angle.


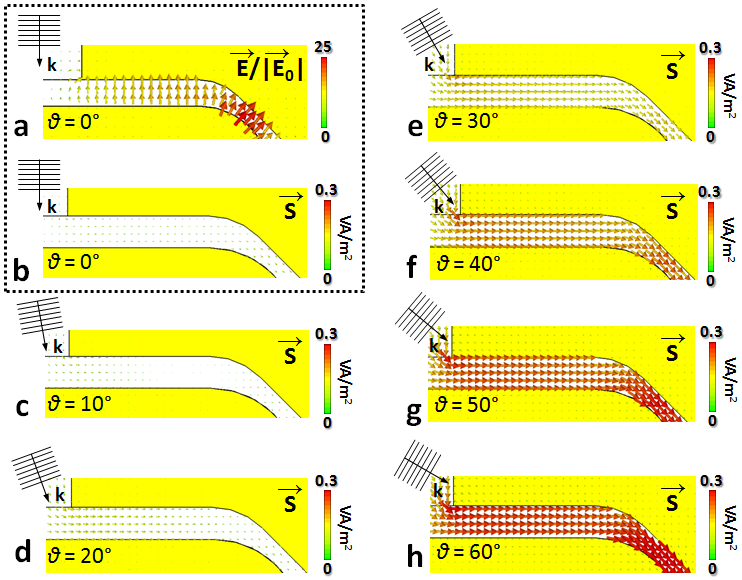


Figure S3. (a) 2D vectorial plot of the normalized electric field distribution associated to the plasmonic mode excited, in normal incidence condition (*θ* = 0°), around *λ* = 700 nm. (b) 2D vectorial plot of the Poynting vector distribution associated to the plasmonic mode of Figure S3(a). (c,d) 2D vectorial plots of the Poynting vector distribution associated to the plasmonic mode of Figure S3(a), for respectively *θ* = 10° and 20°. (e-h) 2D vectorial plots of the Poynting vector distribution associated to *β* mode, in *θ* = 30°, 40°, 50° and 60° incidence condition.

**Section 4: Investigation of *α* and *β* resonances**

In Figures 3(a,b) we show how the electric field associated to both *α* and *β* propagating configurations in SOAs nanocavity is mainly perpendicular to the interfaces between the antennas and the SiO_2_ layer (as confirmed by the upper sketches in Figure S3). In this section we show that the magnetic field $\vec{H}$ associated to these modes is perpendicular to both the electric field $\vec{E}$ and wave vector $\vec{k}$ (see lower sketches in Figure S4).


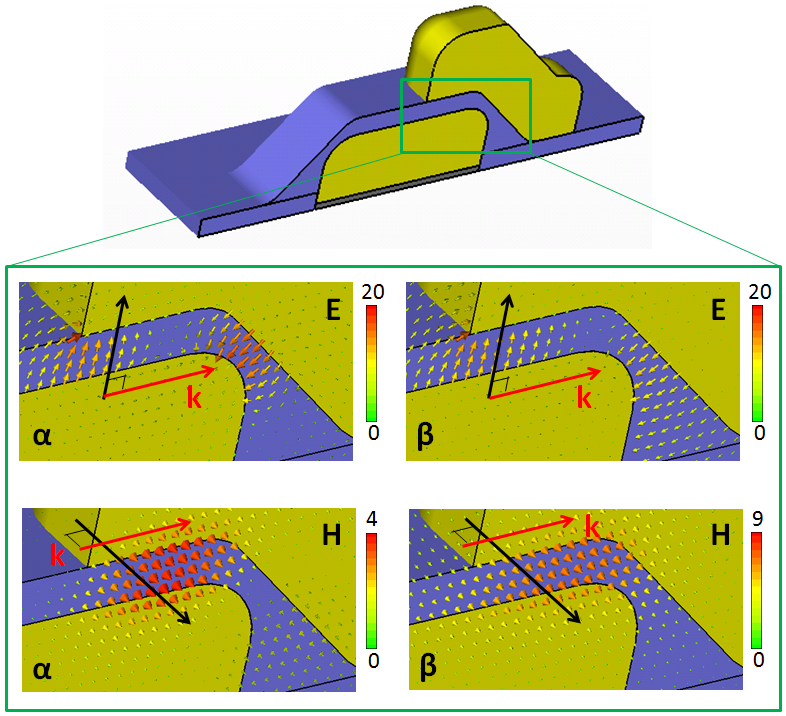


Figure S4. Sketches reporting the electric (upper sketches) and magnetic (lower sketches) field distributions associated to both *α* and *β* propagating configurations on a plane passing through the center of the SOAs assembly and perpendicular to the short axis of the system.

**Section 5: Calculation of the incident power (*P_0_*)**

In the main text, the funneling efficiency *η* of the SOAs system has been defined as the ratio between the power funneled along the cavity *P_t_* and the power *P_0_* impinging on the effective area (defined through the extinction cross-section) of a single SOAs assembly. The last quantity can be expressed as the product between the modulus of the impinging Poynting vector (where *n* is the refractive index of the medium surrounding the SOAs, *ε_0_* the vacuum permeability, *c* the light velocity in vacuum and $\vec{E}_{0}$ the electric field amplitude of the incident plane wave) and the effective interaction surface *Σ_0_*, perpendicular to $\vec{S}_{0}$ and proportional to the SOAs extinction cross section. The morphology of the system implies that *Σ_0_* is dependent on the incidence angle *θ*. However, in order to assume conservative hypothesis, in our work we considered *Σ_0_* constant and equal to the maximum extinction efficiency cross section obtained by the simulative software.


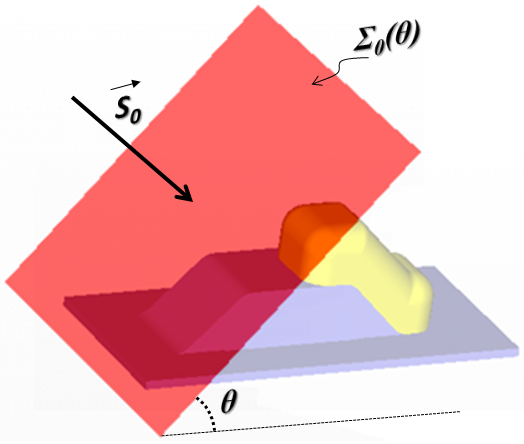


Figure S5. Sketch reporting the impinging Poynting vector $\vec{S}_{0}$ and the surface$\Sigma_{0}$ on which the EM power incident on single SOAs system is calculated.
